# Supplementary material for: Reconstitution of BNIP3/NIX-mitophagy initiation reveals hierarchical flexibility of the autophagy machinery
Source: Nat Cell Biol. 2025 Jul 25;27(8):1272–87. doi: 10.1038/s41556-025-01712-y (PMC12339401; doi:10.1038/s41556-025-01712-y)
Supplement: Supplementary file 1 — Supplementary Fig. 1 [file 41556_2025_1712_MOESM1_ESM.pdf]

nature cell biology

Supplementary information https://doi.org/10.1038/s41556-025-01712-y

Reconstitution of BNIP3/NIX-mitophagy initiation reveals hierarchical flexibility of the autophagy machinery

In the format provided by the authors and unedited

Supplementary information

FACS Gating strategy for DFP-induced mitophagy

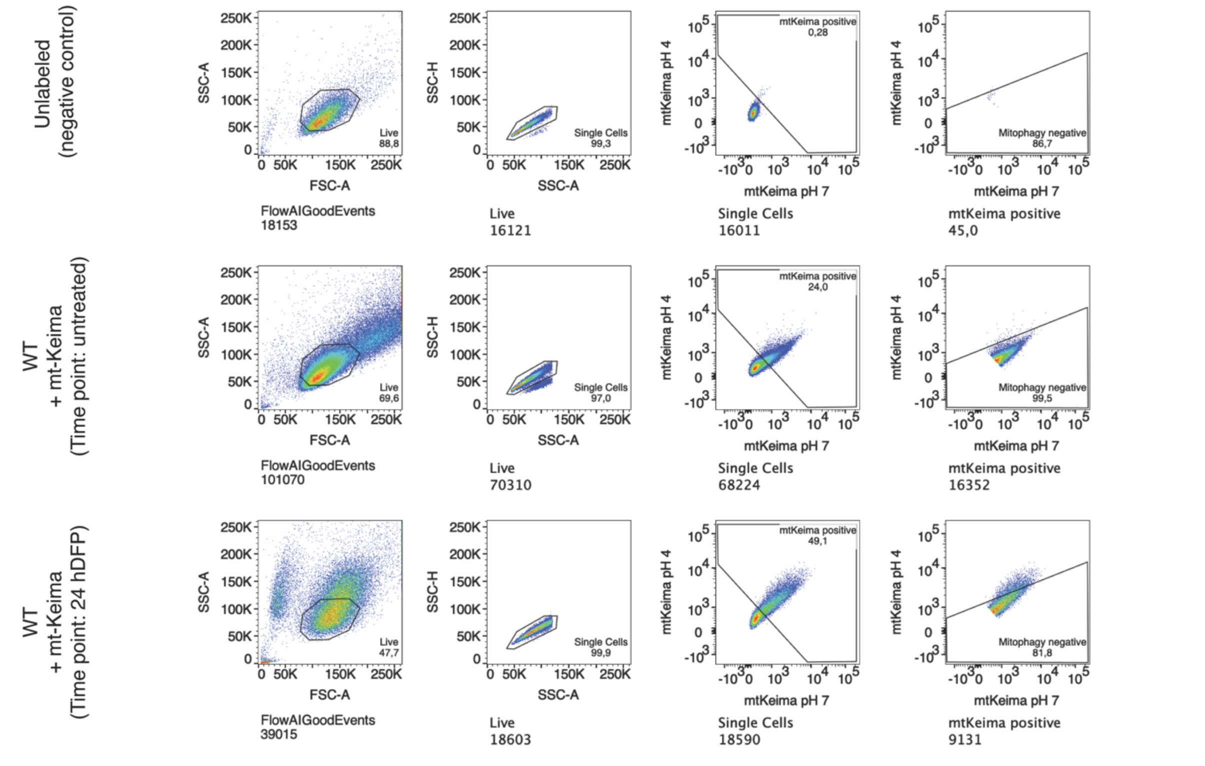

FACS Gating strategy for Rapalog-induced mitophagy

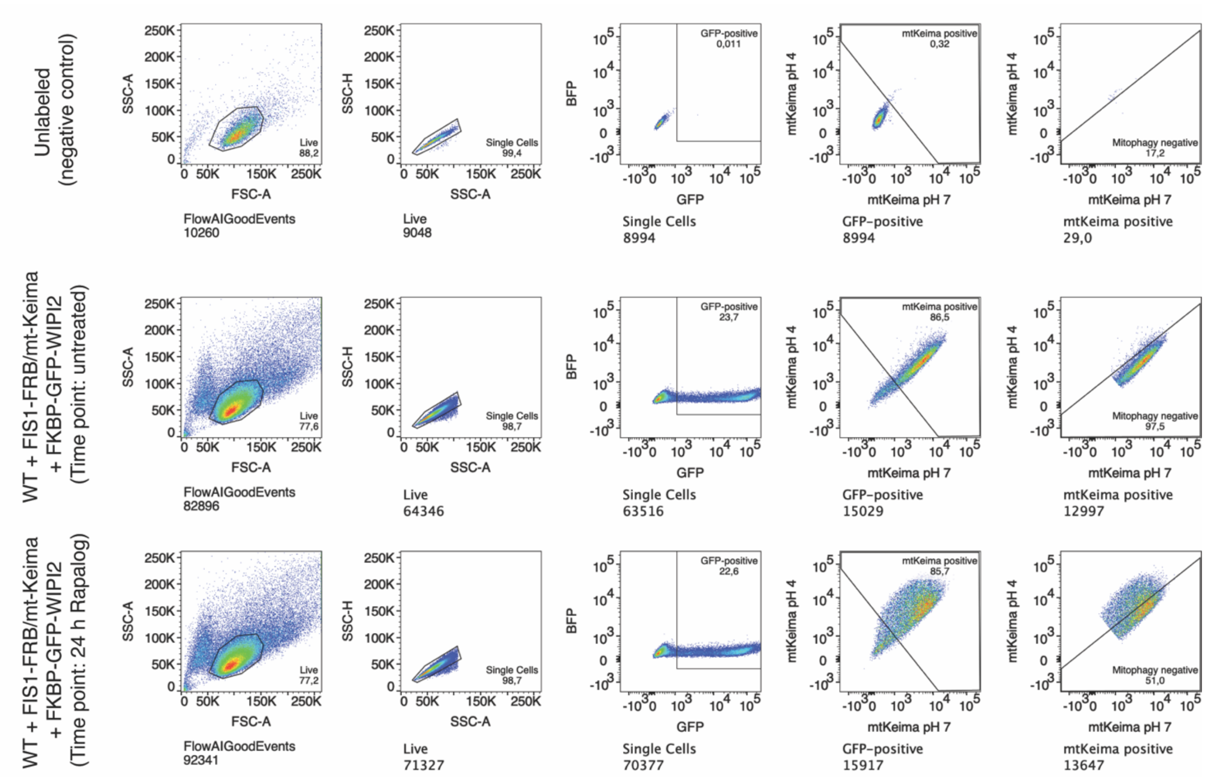

Supplementary Figure 1

Gating strategy for FACS experiments where cells were either treated with DFP or Rapalog. Unstained negative controls are shown. The numbers below each gate represent the number of cells that were gated from the previous plot into the current plot.
