# Supplementary material for: Reconstitution of BNIP3/NIX-mitophagy initiation reveals hierarchical flexibility of the autophagy machinery
Source: Nat Cell Biol. 2025 Jul 25;27(8):1272–87. doi: 10.1038/s41556-025-01712-y (PMC12339401; doi:10.1038/s41556-025-01712-y)
Supplement: Supplementary file 2 — Reporting Summary [file 41556_2025_1712_MOESM2_ESM.pdf]

nature portfolio

Corresponding author(s): Elias Adriaenssens
Sascha Martens

Last updated by author(s): 14/4/25

Reporting Summary

Nature Portfolio wishes to improve the reproducibility of the work that we publish. This form provides structure for consistency and transparency in reporting. For further information on Nature Portfolio policies, see our Editorial Policies and the Editorial Policy Checklist.

Statistics

For all statistical analyses, confirm that the following items are present in the figure legend, table legend, main text, or Methods section.

n/a	Confirmed	
☐	☒	The exact sample size (*n*) for each experimental group/condition, given as a discrete number and unit of measurement
☐	☒	A statement on whether measurements were taken from distinct samples or whether the same sample was measured repeatedly
☐	☒	The statistical test(s) used AND whether they are one- or two-sided<br>*Only common tests should be described solely by name; describe more complex techniques in the Methods section.*
☒	☐	A description of all covariates tested
☐	☒	A description of any assumptions or corrections, such as tests of normality and adjustment for multiple comparisons
☐	☒	A full description of the statistical parameters including central tendency (e.g. means) or other basic estimates (e.g. regression coefficient) AND variation (e.g. standard deviation) or associated estimates of uncertainty (e.g. confidence intervals)
☐	☒	For null hypothesis testing, the test statistic (e.g. *F*, *t*, *r*) with confidence intervals, effect sizes, degrees of freedom and *P* value noted<br>*Give P values as exact values whenever suitable.*
☒	☐	For Bayesian analysis, information on the choice of priors and Markov chain Monte Carlo settings
☒	☐	For hierarchical and complex designs, identification of the appropriate level for tests and full reporting of outcomes
☒	☐	Estimates of effect sizes (e.g. Cohen's *d*, Pearson's *r*), indicating how they were calculated

Our web collection on statistics for biologists contains articles on many of the points above.

Software and code

Policy information about availability of computer code

Data collection	Confocal microscopy images were collected using ZEN software version 2022 (Carl Zeiss Microscopy, GmbH, Germany) connected to a LSM700 or Zen Blue software (Carl Zeiss Microscopy, GmbH, Germany; RRID:SCR_013672) connected to a LSM900. Alphafold-2 (RRID:SCR_025454), Alphafold Multimer (10.1101/2021.10.04.463034) , and Alphafold-3 (RRID:SCR_025454) were used to predict protein and protein complex structures. MD simulations were performed with Gromacs (versions 2023.3 and 2023.4; RRID:SCR_014565) with amber-disp force field. Code for MD simulations can in part be found here: https://github.com/bio-phys/nix-lir-binding-to-wipi2d
Data analysis	1. FlowJo10 (version 10.9.0) software (Tree Star Inc., Ashland, OR, USA) for FACS-data analysis (RRID:SCR_008520).<br>2. PRISM 9 software (version 9.5.1; Graphpad Software, La Jolla, CA, USA) for statistical analysis and generating graphs (RRID:SCR_005375).<br>3. ImageJ software (Schindelin et al. 2015) for immunofluorescence microscopy image analysis (RRID:SCR_003070).<br>4. FACSDiva software (BD FACSDiva software) for flow cytometry experiments (RRID:SCR_001456).<br>5. FreeStyle 1.7 software (Thermo Scientific) for mass spectrometry data.<br>6. Fragpipe (version 19.1 and 20.0) for mass spectrometry data analysis (RRID:SCR_022864) - https://github.com/Nesvilab/FragPipe<br>7. MSFragger (version 3.7 and 3.8) for mass spectrometry data analysis - https://github.com/Nesvilab/MSFragger<br>8. IonQuant (version 1.8.10 and 1.9.8) for mass spectrometry data analysis - https://github.com/Nesvilab/IonQuant<br>9. Philosopher (version 4.8.0 and 5.0.0) for mass spectrometry data analysis - https://github.com/Nesvilab/philosopher<br>10. MaxQuant software version 1.6.17.0 for mass spectrometry data analysis.<br>11. Spectronaut (version 18.3 or 19.5, Biognosys) for mass spectrometry data analysis.<br>12. MSReport (version 0.019 or 0.0.27) for mass spectrometry data analysis.<br>13. LIMMA (version 3.52.1) for statistical analysis of mass spectrometry data.<br>14. Common laboratory contaminants database (developed in house; https://github.com/maxperutzlabs-ms/perutz-ms-contaminants).<br>15. ChimeraX-1.8 for visualisation of predicted protein structures (RRID:SCR_015872).

16. Python3 (version 3.10.9) for MD analysis (RRID:SCR_008394).
17. Anaconda3 (version 2019.10) for MD analysis (RRID:SCR_025572).
18. iPython (version 8.10.0) for MD analysis (RRID:SCR_001658).
19. Numpy (version 1.26.2) for MD analysis (RRID:SCR_008633).
20. Matplotlib (version 3.7.1) for MD analysis (RRID:SCR_008624).
21. MDAnalysis (version 2.3.0) for MD analysis (RRID:SCR_025610).
22. VMD (version 1.9.3 and 1.9.4) for MD analysis (RRID:SCR_001820).
23. LINCS algorithm to describe hydrogen bonds during MD analysis
24. Gromacs (version 2023.3 and 2023.4) for MD analysis ((RRID:SCR_014565)).
25. Airyscan processing plug-in in Zen Blue software (Zeiss)
26. Huygens Professional 24.04 (Scientific Volume Imaging) was used to deconvolve LSM700 images (RRID:SCR_014237)
27. Synthego ICE CRISPR analysis (version 2) (RRID:SCR_024508) https://www.synthego.com/products/bioinformatics/crispr-analysis

For manuscripts utilizing custom algorithms or software that are central to the research but not yet described in published literature, software must be made available to editors and reviewers. We strongly encourage code deposition in a community repository (e.g. GitHub). See the Nature Portfolio guidelines for submitting code & software for further information.

Data

Policy information about availability of data

All manuscripts must include a data availability statement. This statement should provide the following information, where applicable:

- Accession codes, unique identifiers, or web links for publicly available datasets
- A description of any restrictions on data availability
- For clinical datasets or third party data, please ensure that the statement adheres to our policy

Raw files associated with this work have been made available on Zenodo (https://doi.org/10.5281/zenodo.14867723). The mass spectrometry proteomics data have been deposited to the ProteomeXchange Consortium via the PRIDE partner repository with the dataset identifiers PXD060351, PXD060356, and PXD060363. Source data are provided with this paper. Plasmids constructed for and used in this manuscript are available at Addgene. The data, protocols, and key lab materials used in this study are listed in a Key Resource Table alongside their persistent identifiers as Supplementary file. All other data supporting the findings of this study are available from the corresponding author on reasonable request. Further information on the research design is available in the Nature Research Reporting Summary linked to this article.

Zenodo: https://doi.org/10.5281/zenodo.14867723
Github MD analysis: https://github.com/bio-phys/nix-lir-binding-to-wipi2d
Pride (MS datasets): PXD060351, PXD060356, and PXD060363

Research involving human participants, their data, or biological material

Policy information about studies with human participants or human data. See also policy information about sex, gender (identity/presentation), and sexual orientation and race, ethnicity and racism.

Reporting on sex and gender	N/A
Reporting on race, ethnicity, or other socially relevant groupings	N/A
Population characteristics	N/A
Recruitment	N/A
Ethics oversight	N/A

Note that full information on the approval of the study protocol must also be provided in the manuscript.

Field-specific reporting

Please select the one below that is the best fit for your research. If you are not sure, read the appropriate sections before making your selection.

☒ Life sciences ☐ Behavioural & social sciences ☐ Ecological, evolutionary & environmental sciences

For a reference copy of the document with all sections, see nature.com/documents/nr-reporting-summary-flat.pdf

Life sciences study design

All studies must disclose on these points even when the disclosure is negative.

Sample size	No statistical methods were applied to pre-evaluate sample size. Experiments were performed at least as three replicates, according to current practices in the field. Statistical analysis was performed on experiments for which the sample size included at least 3 biological replicates. Sample sizes were based on previous experience and current standards in the field.

Data exclusions	No data were excluded from the analyses.
Replication	All experiments were replicated at least three times with similar findings. Samples sizes are provided in the figure legends.
Randomization	Samples were allocated into experimental groups by genotype of knockout condition. Covariates were controlled for by maintaining all samples in the same growth and media conditions.
Blinding	The investigators were not blinded to treatment or genotype allocations during this study. For cell based and biochemistry experiments, it was not possible to blind the experimenter.

Reporting for specific materials, systems and methods

We require information from authors about some types of materials, experimental systems and methods used in many studies. Here, indicate whether each material, system or method listed is relevant to your study. If you are not sure if a list item applies to your research, read the appropriate section before selecting a response.

Materials & experimental systems

n/a	Involved in the study
☐	☒ Antibodies
☐	☒ Eukaryotic cell lines
☒	☐ Palaeontology and archaeology
☒	☐ Animals and other organisms
☒	☐ Clinical data
☒	☐ Dual use research of concern
☒	☐ Plants

Methods

n/a	Involved in the study
☒	☐ ChIP-seq
☐	☒ Flow cytometry
☒	☐ MRI-based neuroimaging

Antibodies

Antibodies used

anti-α-Tubulin (1:5000, Abcam Cat# ab7291, RRID:AB_2241126)
anti-ATG13 (1:1000 for western blot, Cell Signaling Technology Cat# 13468, RRID:AB_2797419)
anti-ATG13 (1:200 for immunofluorescence, Cell Signaling Technology, Cat# 13468; RRID:AB_2797419)
anti-Beclin1 (1:1000, Cell Signaling Technology Cat# 3738, RRID:AB_490837)
anti-phospho-Beclin1 Ser30 (1:1000, Cell Signaling Technology Cat# 54101, RRID:AB_3102019)
anti-BNIP3 (1:1000, Cell Signaling Technology Cat# 44060, RRID:AB_2799259)
anti-CCPG1 (1:1000, Cell Signaling Technology Cat# 80158, RRID:AB_2935809)
anti-COXII (1:1000, Abcam Cat# ab110258, RRID:AB_10887758)
anti-COXII (1:1000, Cell Signaling Technology Cat# 31219, RRID:AB_2936222)
anti-4EBP1 (1:1000, Proteintech Cat# 60246-1-Ig, RRID:AB_2881368)
anti-FIP200 (1:1000, Cell Signaling Technology Cat# 12436, RRID:AB_2797913)
anti-GFP (1:1000, Millipore Cat# MABC1689, RRID:AB_3675504)
anti-GST (1:1000, Sigma-Aldrich, SAB4200237 , RRID: AB_2858197)
anti-penta-His (1:1000, Qiagen Cat# 34660, RRID:AB_2619735)
anti-LC3B (1:500, Nanotools Cat# 0260-100/LC3-2G6, RRID:AB_2943418)
anti-NIX/BNIP3L (1:1000, Cell Signaling Technology Cat# 12396, RRID:AB_2688036)
anti-OPTN (1:500, Sigma Aldrich Cat# HPA003279, RRID:AB_1079527)
anti-HSP60 (1:800, Abcam Cat# ab46798, RRID:AB_881444)
anti-phospho-OPTN Ser177 (1:1000, Cell Signaling Technology Cat# 57548, RRID:AB_2799529)
anti-p62/SQSTM1 (1:1000, Abnova Cat# H00008878-M01, RRID:AB_437085)
anti-phospho-p62/SQSTM1 Ser403 (1:1000, Cell Signaling Technology Cat# 39786, RRID:AB_2799162)
anti-PPTC7 (1:1000, Abcam, ab122548, RRID: AB_11127117)
anti-TEX264 (1:1000, Sigma-Aldrich Cat# HPA017739, RRID:AB_1857910)
anti-ULK1 (1:1000, Cell Signaling Technology Cat# 8054, RRID:AB_11178668)
anti-V5 (1:1000, Thermo Fisher Scientific Cat# R960-25, RRID:AB_2556564)
anti-WIPI1 (1:200, Santa Cruz Biotechnology, Cat# sc-376205, RRID:AB_10989262)
anti-WIPI2 (1:1000 for western blot, Bio-Rad Cat# MCA5780GA, RRID:AB_ 10845951)
anti-WIPI2 (1:100 for immunofluorescence, Abcam Cat# ab105459, RRID:AB_10860881)
anti-WIPI3 (1:200, Santa Cruz Biotechnology, sc-514194, RRID:AB_3101990)
anti-WIPI4 (1:1000, Abcam, ab168532; RRID:AB_3101989)

HRP conjugated polyclonal goat anti-mouse (Jackson ImmunoResearch Labs Cat# 115-035-003, RRID:AB_10015289)
HRP conjugated polyclonal goat anti-rabbit (Jackson ImmunoResearch Labs Cat# 111-035-003, RRID:AB_2313567)
AlexaFluor-488 goat anti-Mouse IgG (H+L) (1:500, Thermo Fisher Scientific Cat# A-11001, RRID:AB_2534069)
AlexaFluor-546 goat anti-rabbit IgG (H+L) (1:500, Thermo Fisher, Cat# A-11035; RRID: AB_2534093).

Validation

Antibodies were selected based on their use in other publications and/or validation by the manufacturers for their respective application. Where possible, knockout cell lines were used to validate the specificity of the antibodies further.

Eukaryotic cell lines

Policy information about cell lines and Sex and Gender in Research

Cell line source(s)	All parental cell lines (HeLa, HEK293T, HEK293F) were acquired from the American Type Culture Collection (ATCC). HeLa knockout cell lines were generated during this study and submitted to Cellosaurus. HAP1 cells (RRID:CVCL_Y019) were acquired from Horizon Discovery. Sf9 insect cells were acquired from Thermo Fisher (12659017, RRID:CVCL_0549).
Authentication	Authentication was performed upon first arrival in the lab based on morphology according to ATCC.
Mycoplasma contamination	All cell lines were routinely tested for mycoplasma contamination. All cell lines were negative throughout the study.
Commonly misidentified lines (See ICLAC register)	The cell lines used in this study are not listed as commonly misidentified cell lines. This was verified in the ICLAC table of commonly misidentified cell lines.

Plants

Seed stocks	N/A
Novel plant genotypes	N/A
Authentication	N/A

Flow Cytometry

Plots

Confirm that:

☒ The axis labels state the marker and fluorochrome used (e.g. CD4-FITC).

☒ The axis scales are clearly visible. Include numbers along axes only for bottom left plot of group (a 'group' is an analysis of identical markers).

☒ All plots are contour plots with outliers or pseudocolor plots.

☒ A numerical value for number of cells or percentage (with statistics) is provided.

Methodology

Sample preparation	HeLa cells were transduced with lentiviral or retroviral vectors that would express the fluorophore. Cells were treated according the experimental protocol and then collected by removing the medium, washing the cells with 1x PBS (14190169, Thermo Fisher), trypsinisation (T3924, Sigma), and resuspending in complete DMEM medium (41966052, Thermo Fisher). Filtered through 35 µm cell-strainer caps (352235, Falcon) and analysed by an LSR Fortessa Cell Analyzer (BD Biosciences). Lysosomal mt-mKeima was measured using dual excitation ratiometric pH measurements at 405 (pH 7) and 561 (pH 4) nm lasers with 710/50-nm and 610/20-nm detection filters, respectively. Additional channels used for fluorescence compensation were BFP and GFP. Single fluorescence vector expressing cells were prepared to adjust photomultiplier tube voltages to make sure the signal was within detection limits, and to calculate the compensation matrix in BD FACSDiva Software. Depending on the experiment, we gated for BFP-positive, GFP-positive, and mKeima-positive cells with the appropriate compensation. For each sample, 10,000 mKeima-positive events were collected, and data were analyzed in FlowJo (version 10.9.0).
Instrument	LSR Fortessa Cell Analyzer (BD Biosciences)
Software	BD FACSDiva software during data collection and FlowJo10 software (Tree Star Inc., Ashland, OR, USA) for data analysis.
Cell population abundance	Cells were only included when they were viable, single cells (exclusion doublets), and depending on the experiment whether they were GFP-, and/or mt-mKeima positive.
Gating strategy	Gating was optimized, depending on the experiment, for GFP- and/or BFP- and mt-mKeima positive cells after viable singlets were separated from potentially dead cells or doublets based on scatter.

☒ Tick this box to confirm that a figure exemplifying the gating strategy is provided in the Supplementary Information.
