# Supplementary material for: Reconstitution of BNIP3/NIX-mitophagy initiation reveals hierarchical flexibility of the autophagy machinery
Source: Nat Cell Biol. 2025 Jul 25;27(8):1272–87. doi: 10.1038/s41556-025-01712-y (PMC12339401; doi:10.1038/s41556-025-01712-y)
Supplement: Supplementary file 5 — Unprocessed gels [file 41556_2025_1712_MOESM5_ESM.pdf]

Figure 1B

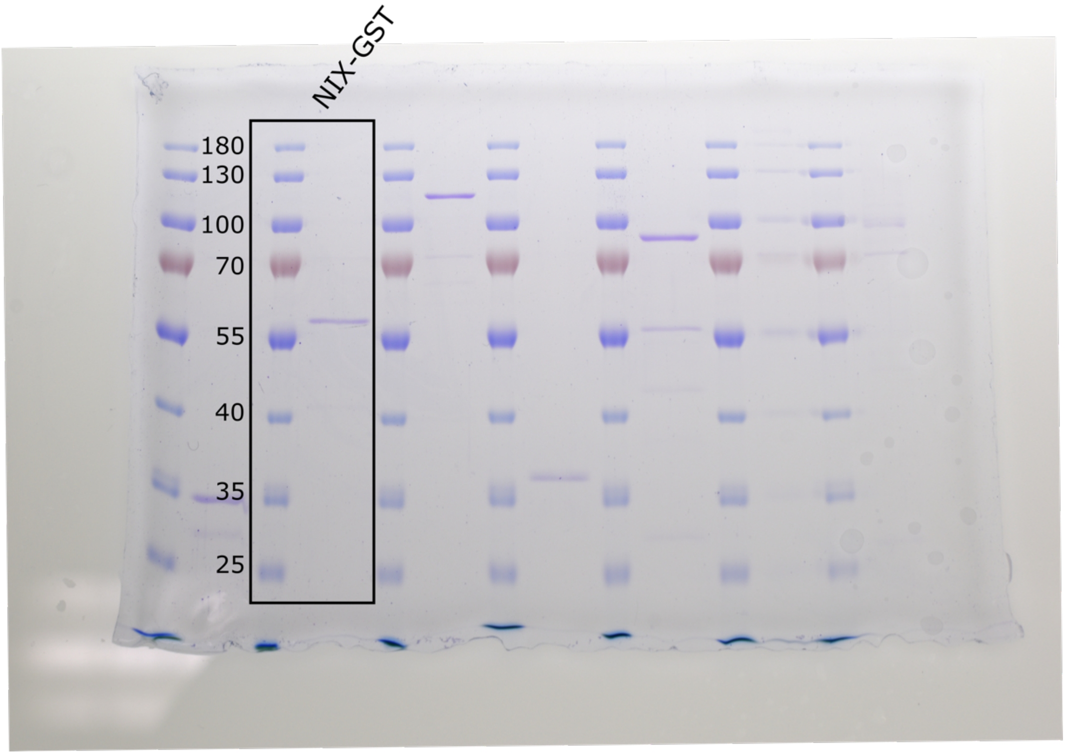
NIX-GST
180
130
100
70
55
40
35
25

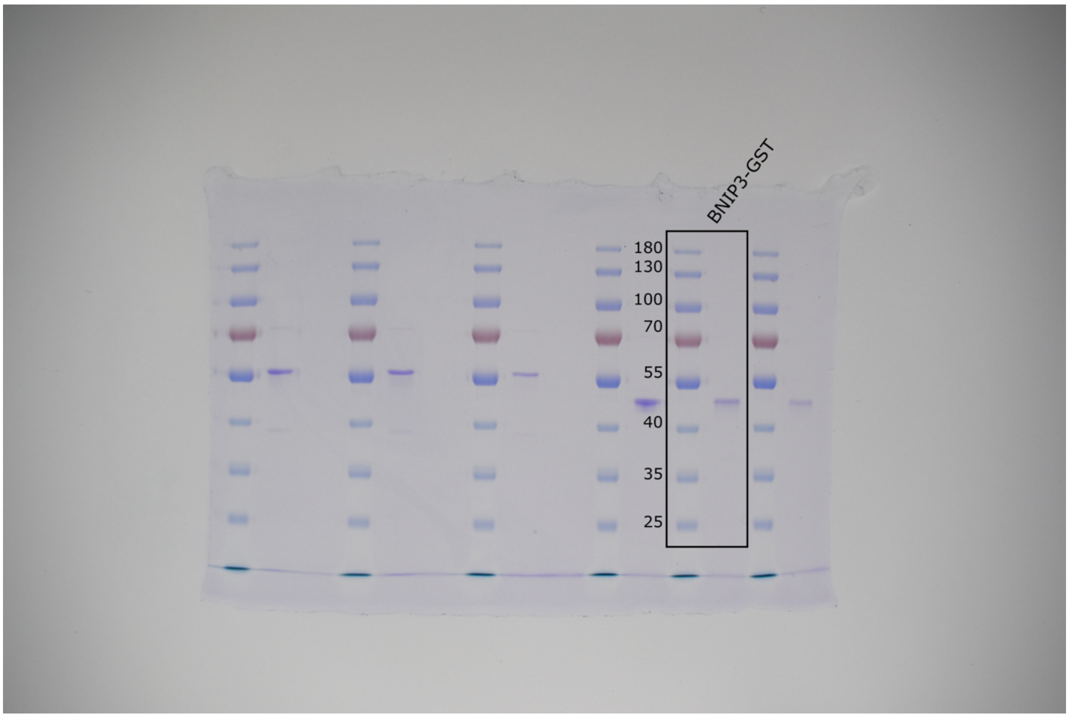
BNIP3-GST
180
130
100
70
55
40
35
25

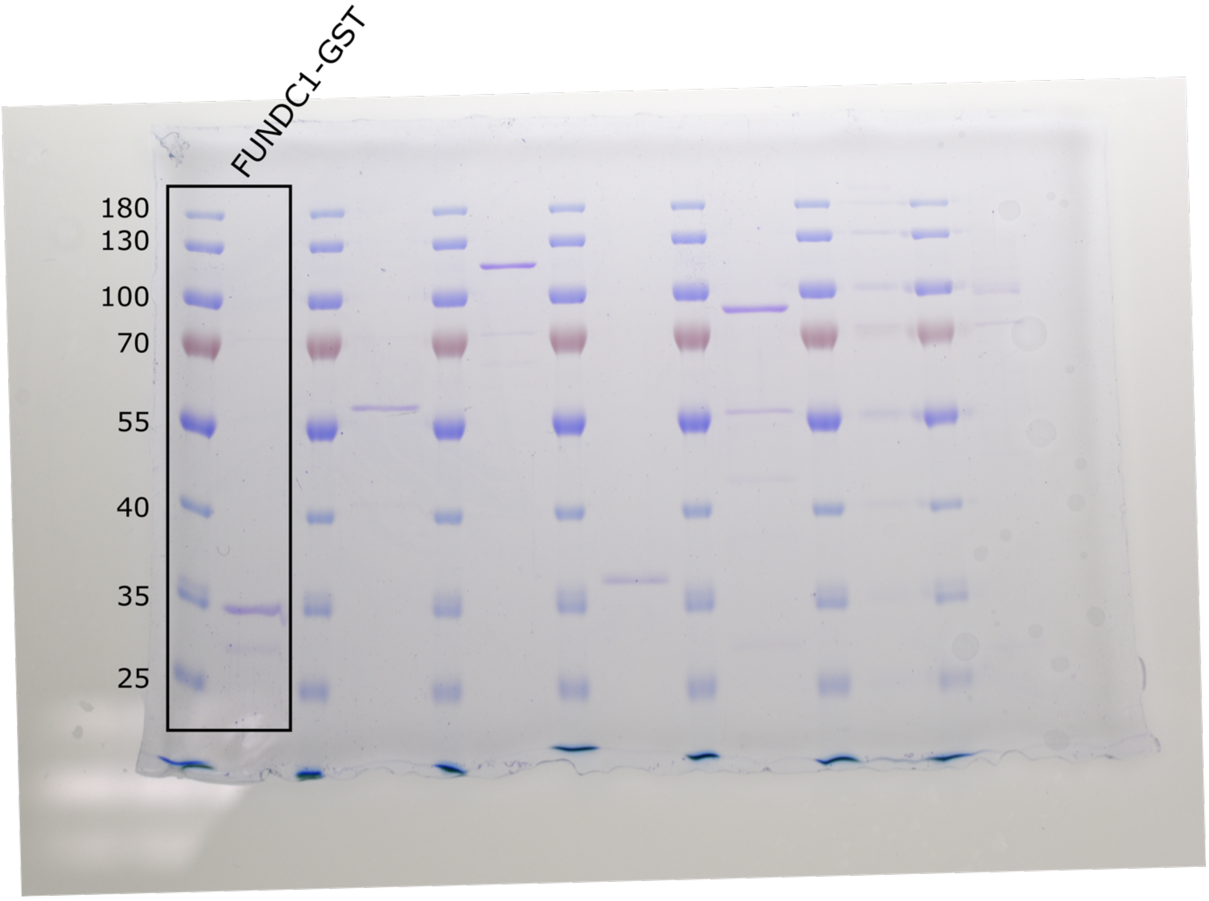
FUNDC1-GST
180
130
100
70
55
40
35
25

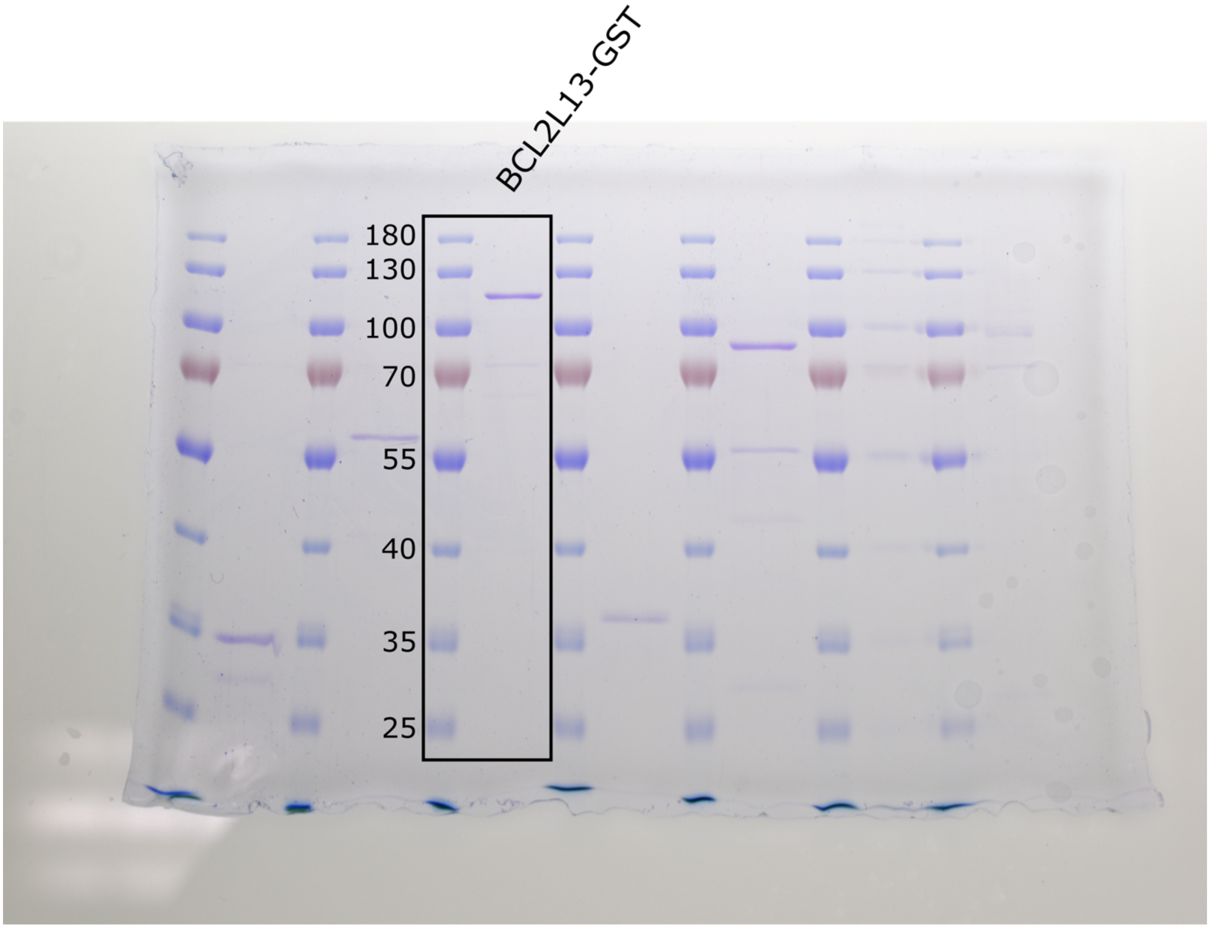
BCL2L13-GST
180
130
100
70
55
40
35
25
