# Supplementary figures and images for: Reconstitution of BNIP3/NIX-mitophagy initiation reveals hierarchical flexibility of the autophagy machinery
Source: Nat Cell Biol. 2025 Jul 25;27(8):1272–87. doi: 10.1038/s41556-025-01712-y (PMC12339401; doi:10.1038/s41556-025-01712-y)

Figure 2B

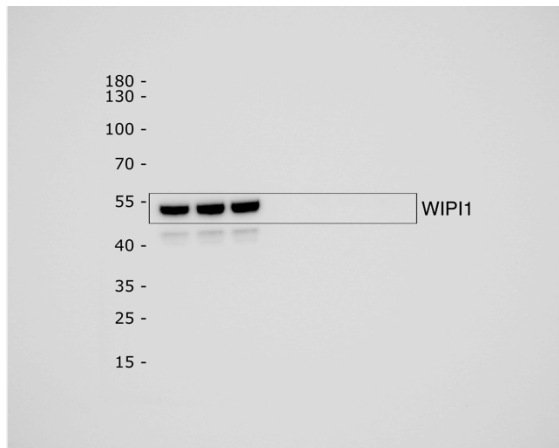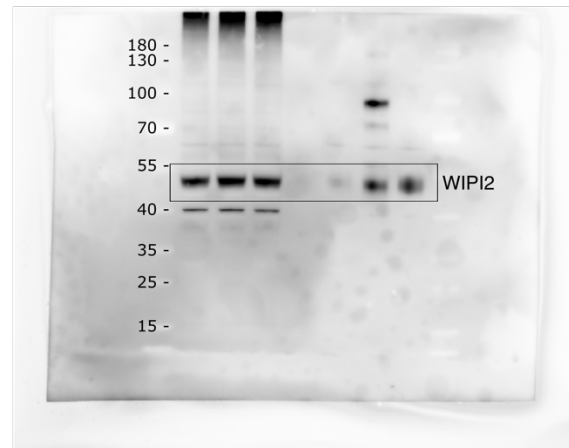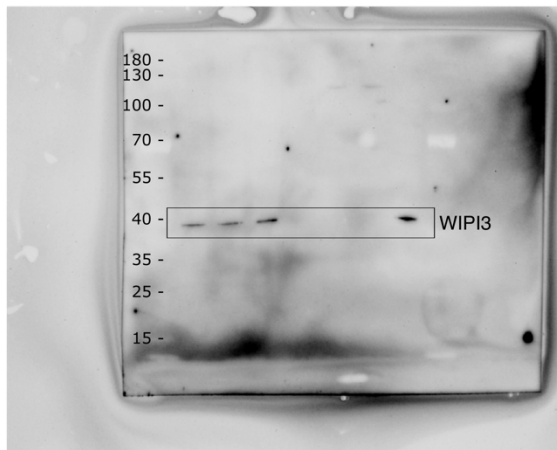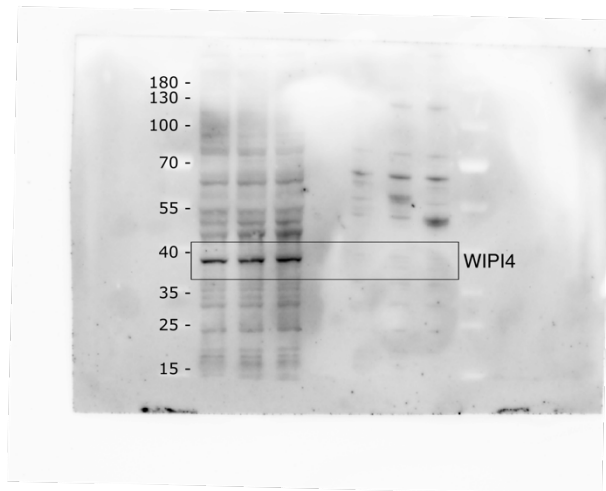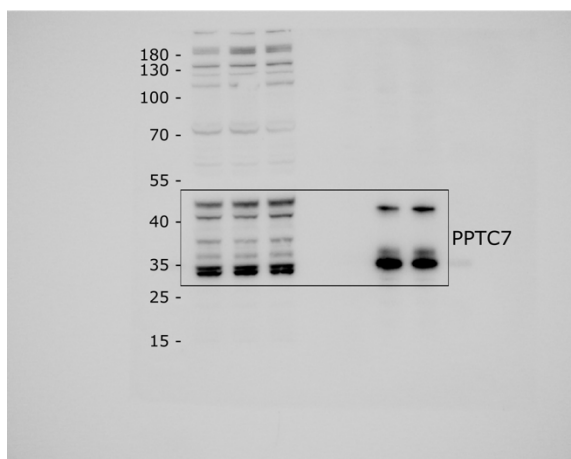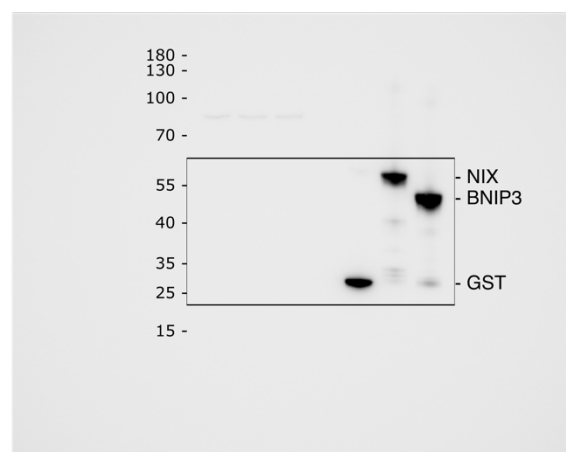

Supplement: Supplementary file 7 — Unprocessed western blots [file 41556_2025_1712_MOESM7_ESM.pdf]

Figure 3E

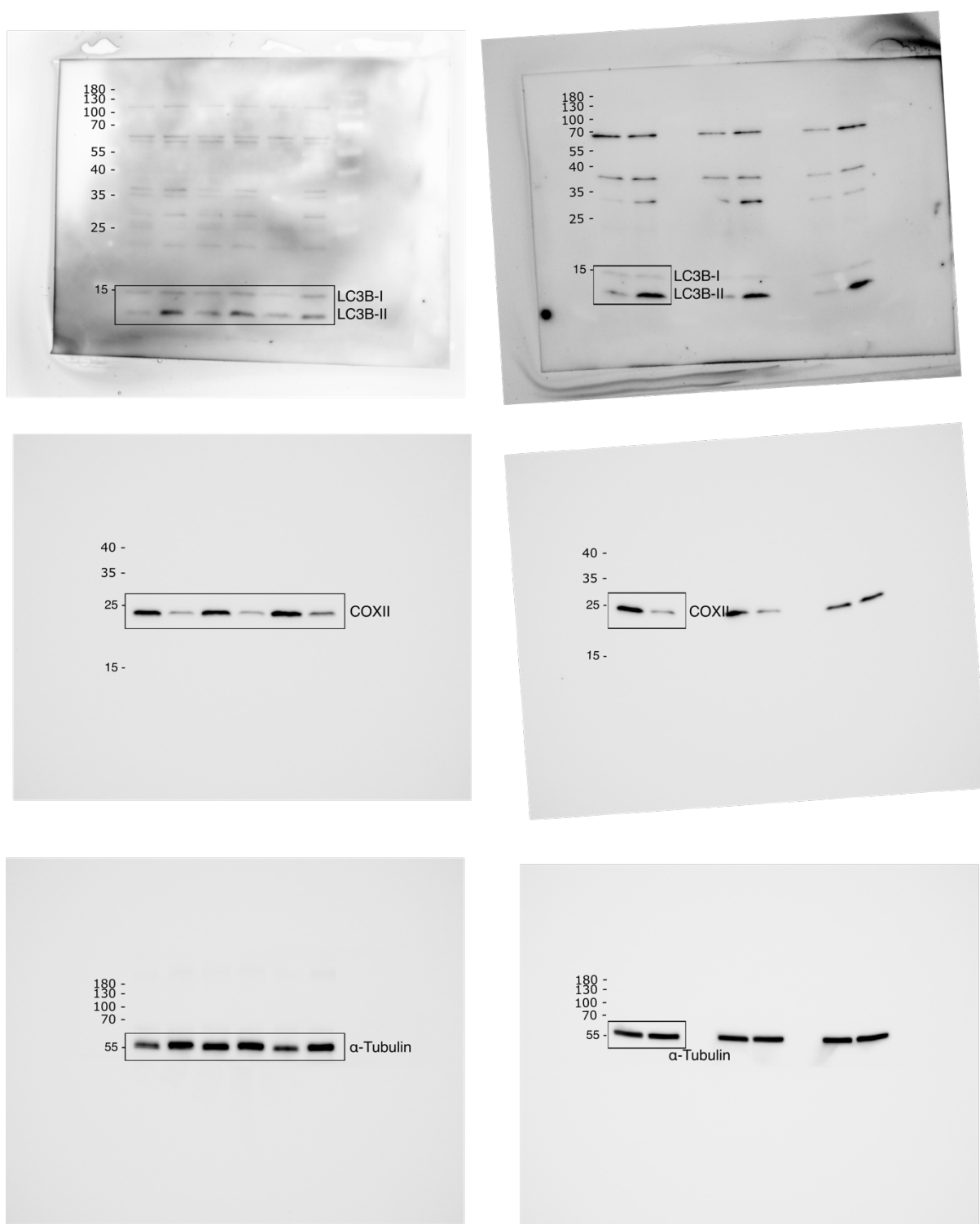

Supplement: Supplementary file 9 — Unprocessed western blots [file 41556_2025_1712_MOESM9_ESM.pdf]

Figure 4B

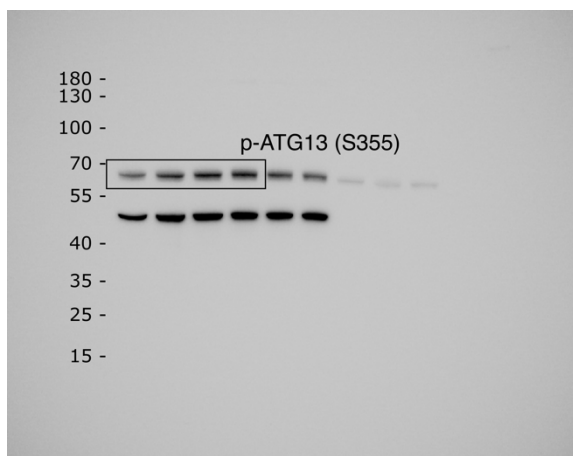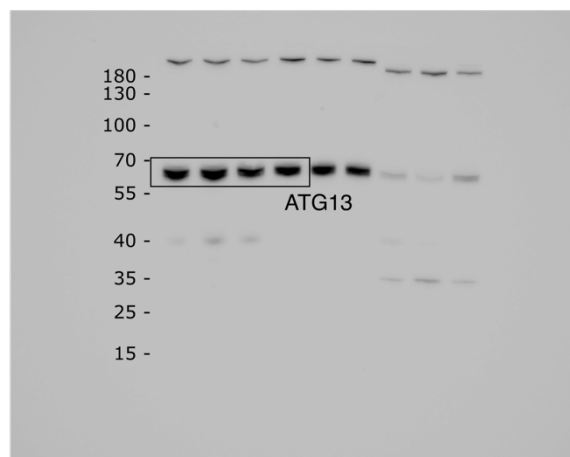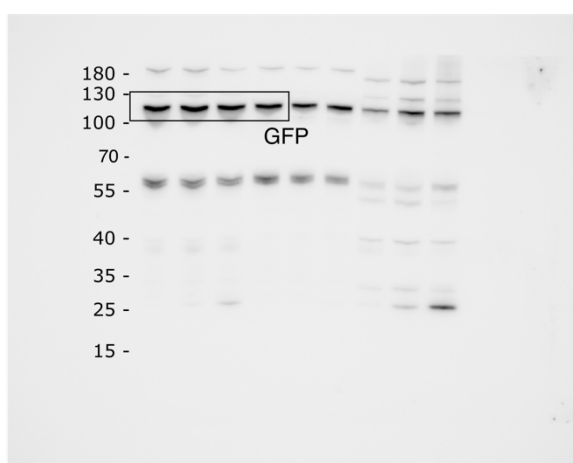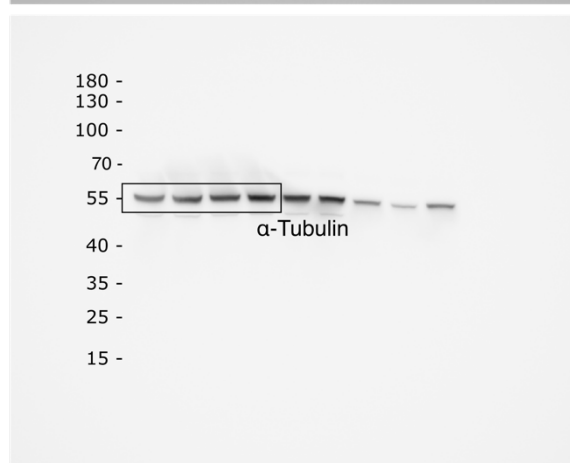

Supplement: Supplementary file 11 — Unprocessed western blots [file 41556_2025_1712_MOESM11_ESM.pdf]

Figure 7A

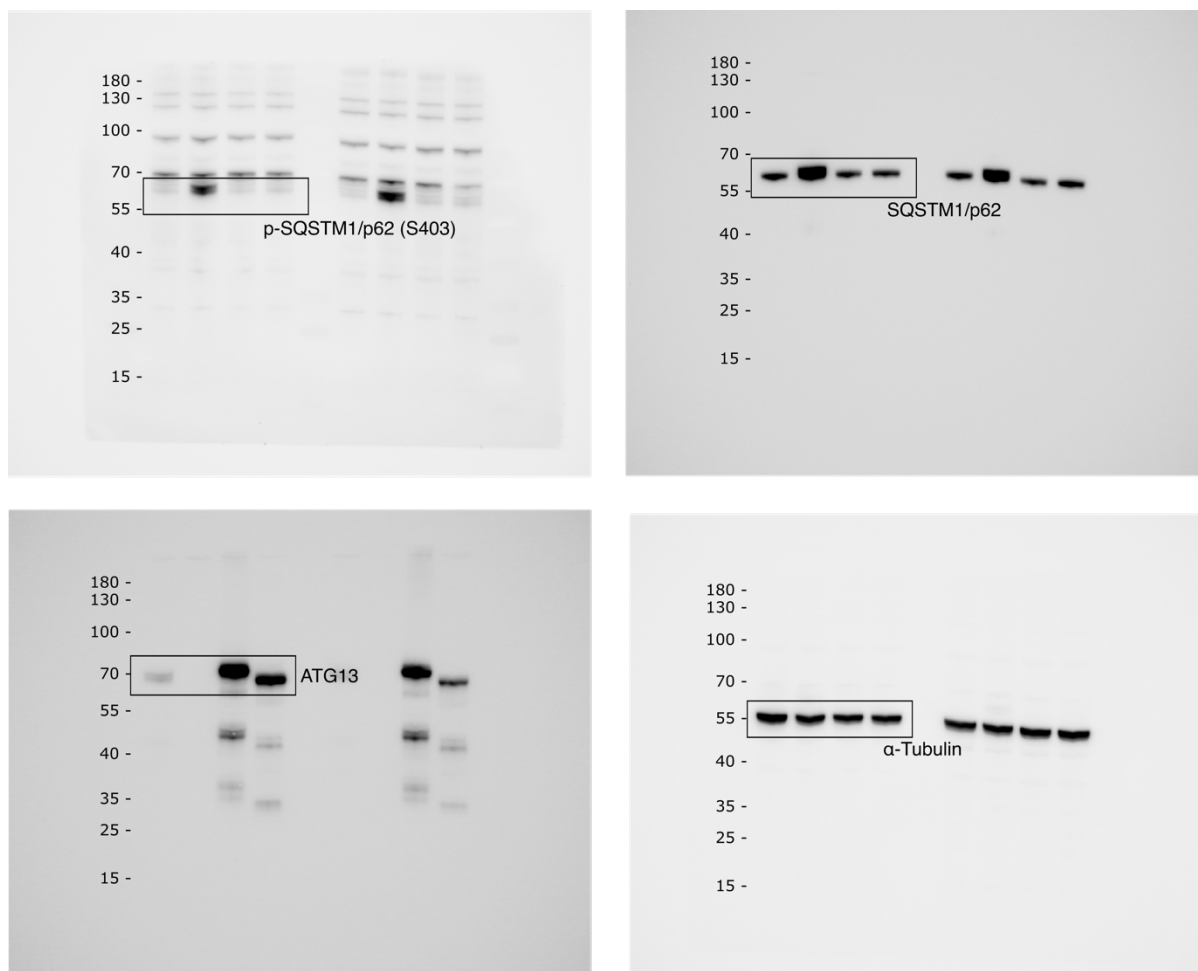

Figure 7B

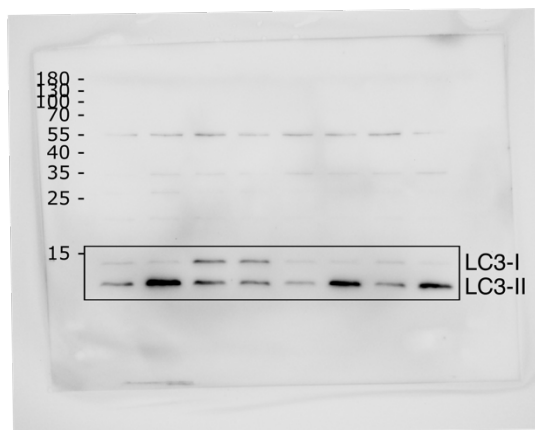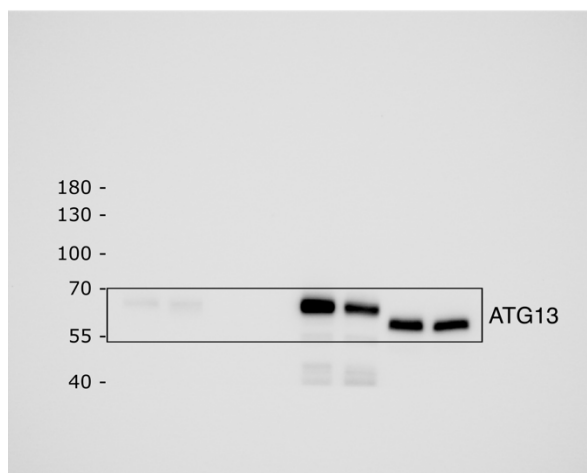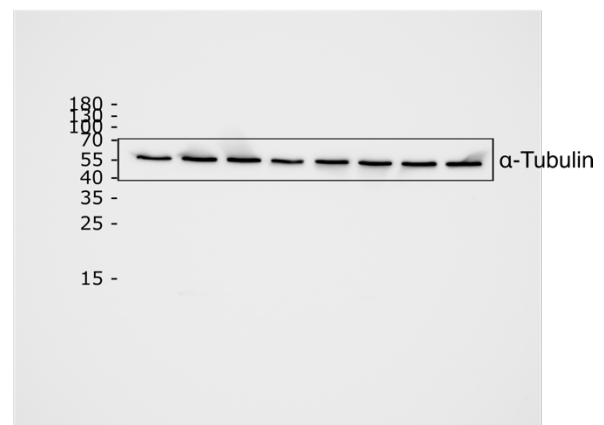

Figure 7E

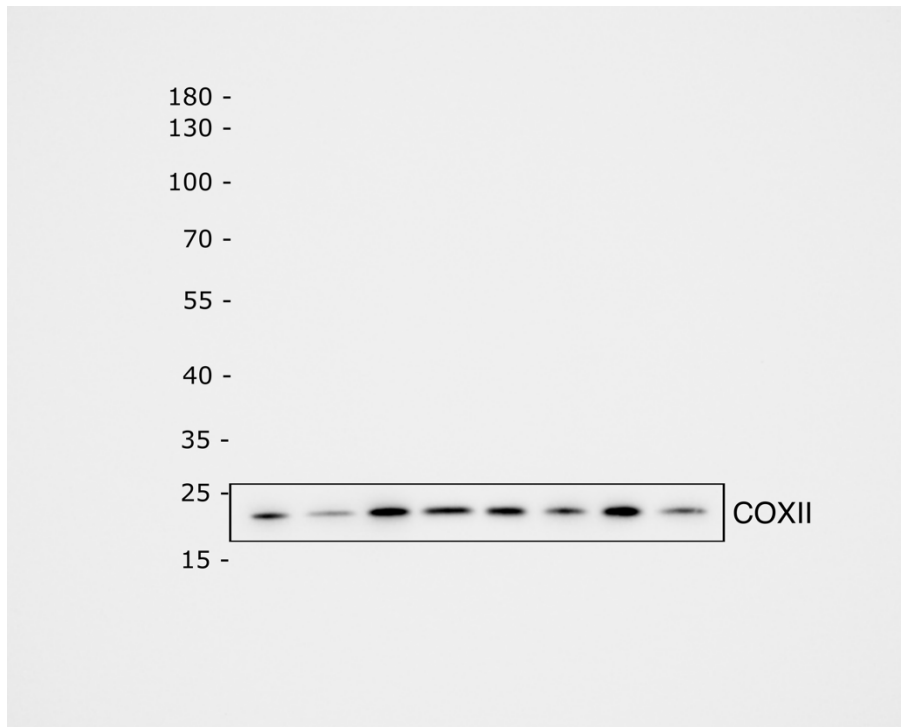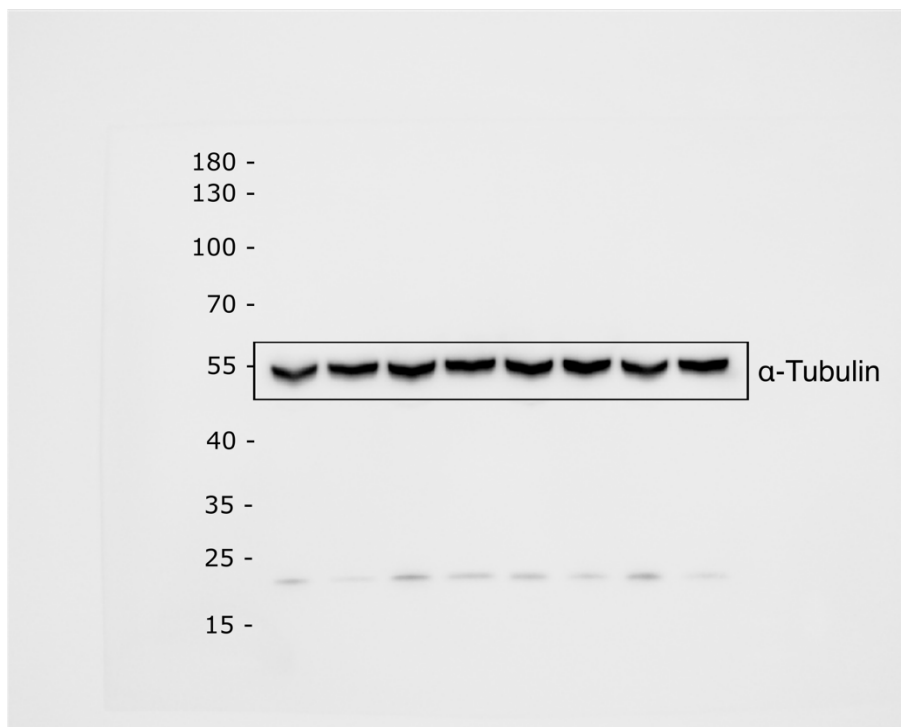

Supplement: Supplementary file 15 — Unprocessed western blots [file 41556_2025_1712_MOESM15_ESM.pdf]

Figure 8F

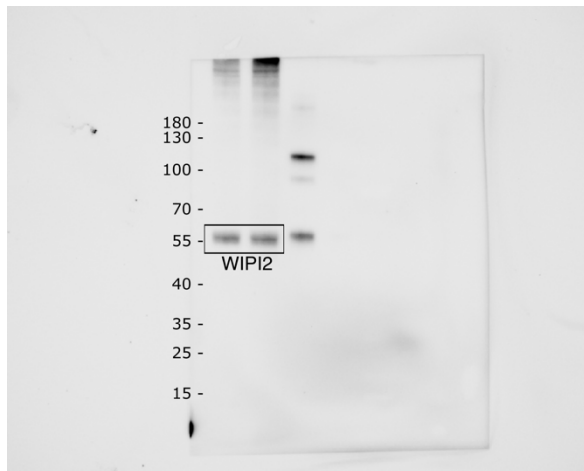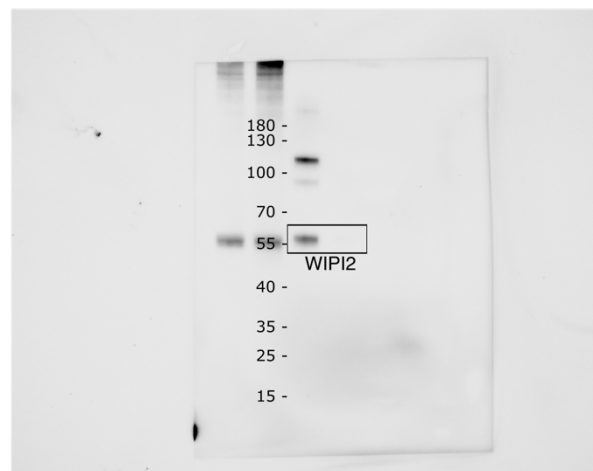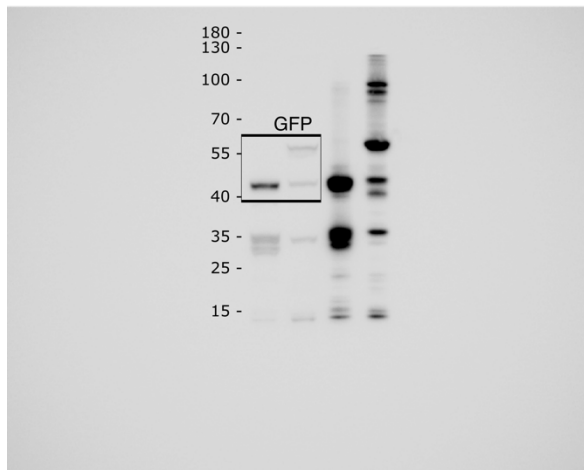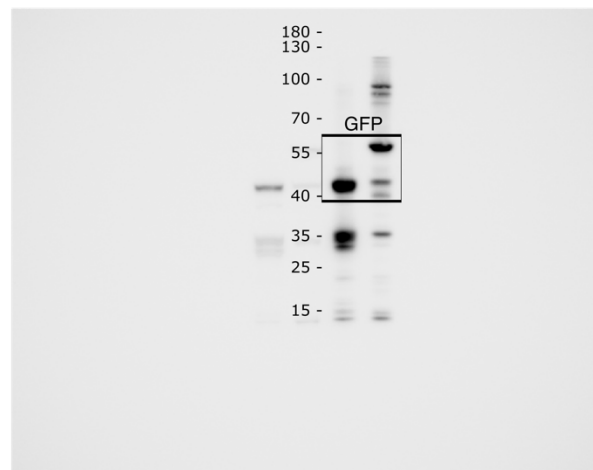

Figure 8G

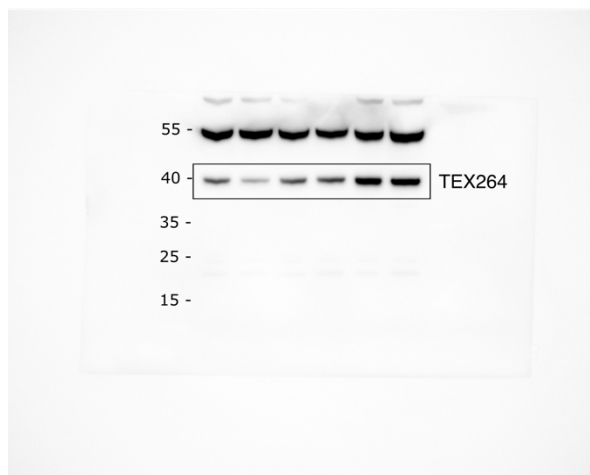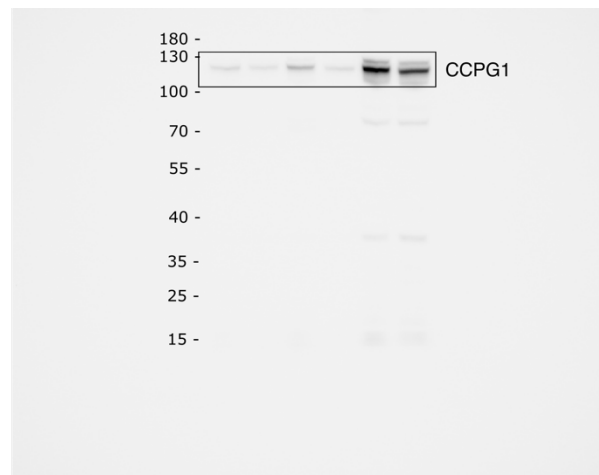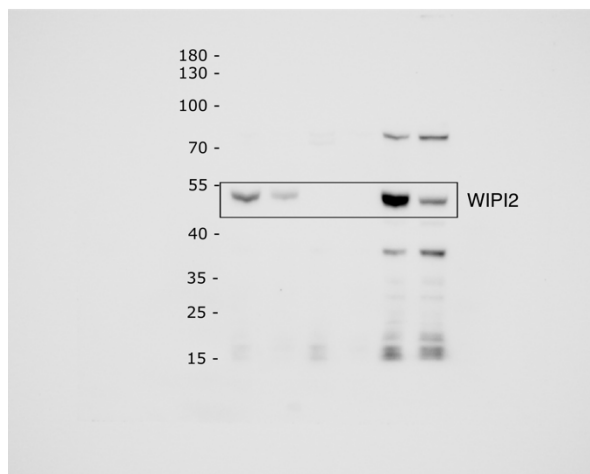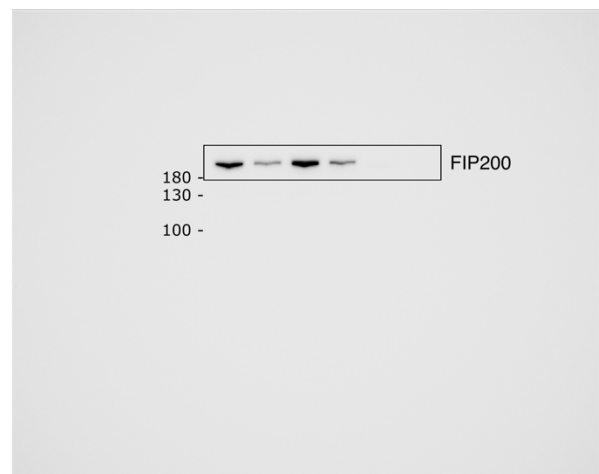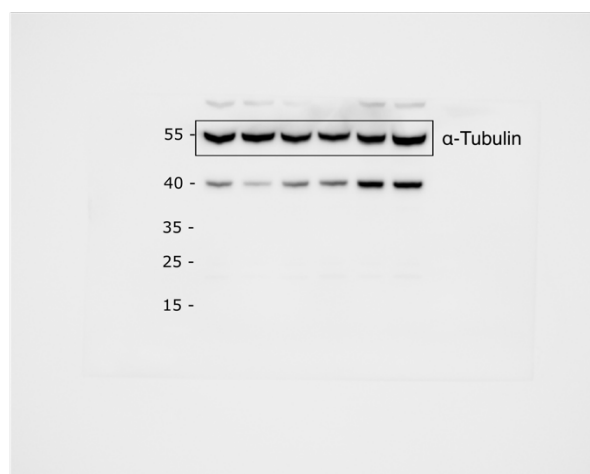

Supplement: Supplementary file 17 — Unprocessed western blots [file 41556_2025_1712_MOESM17_ESM.pdf]

Figure S2A

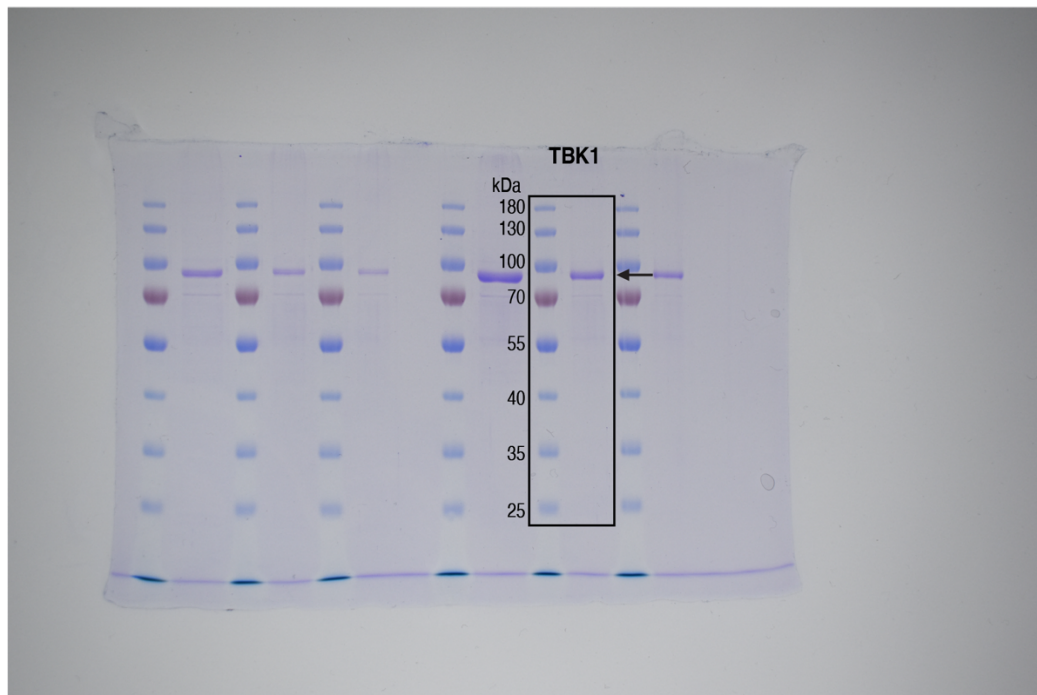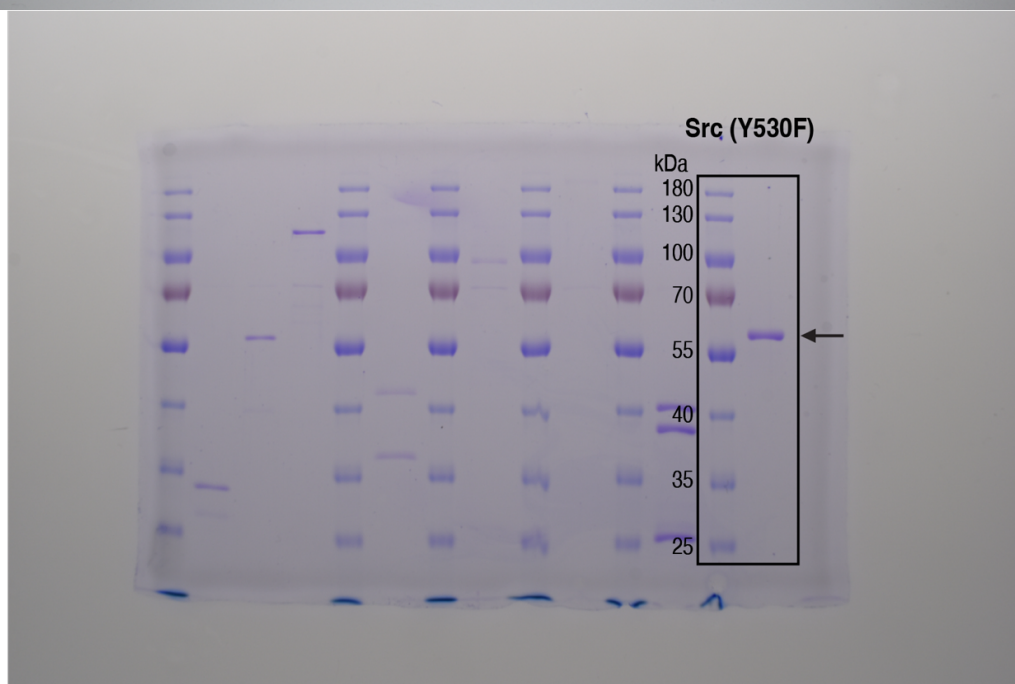

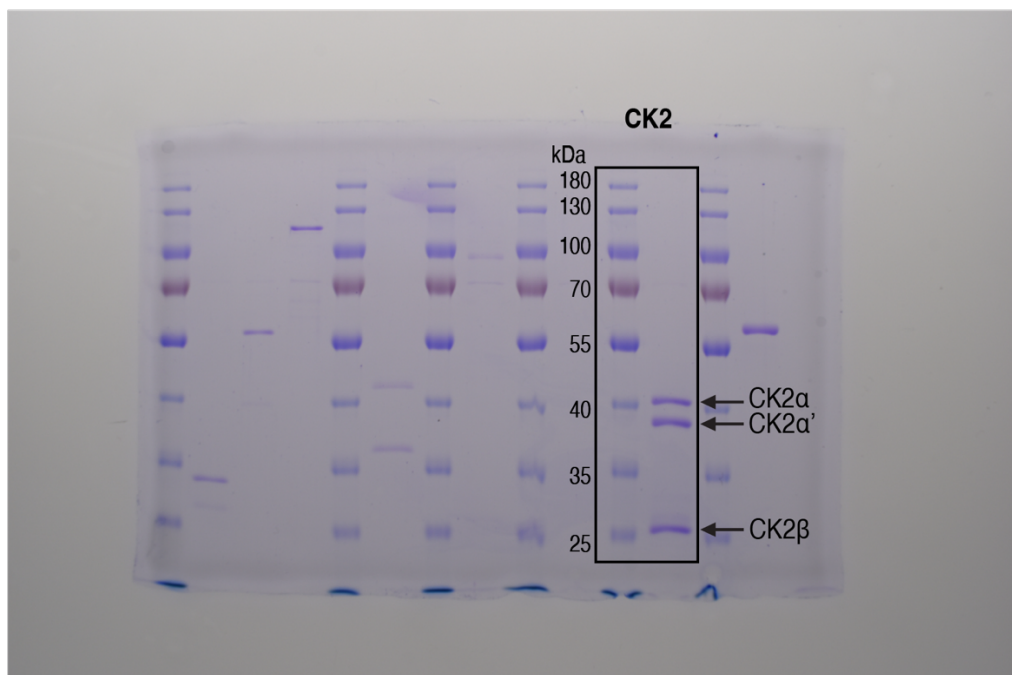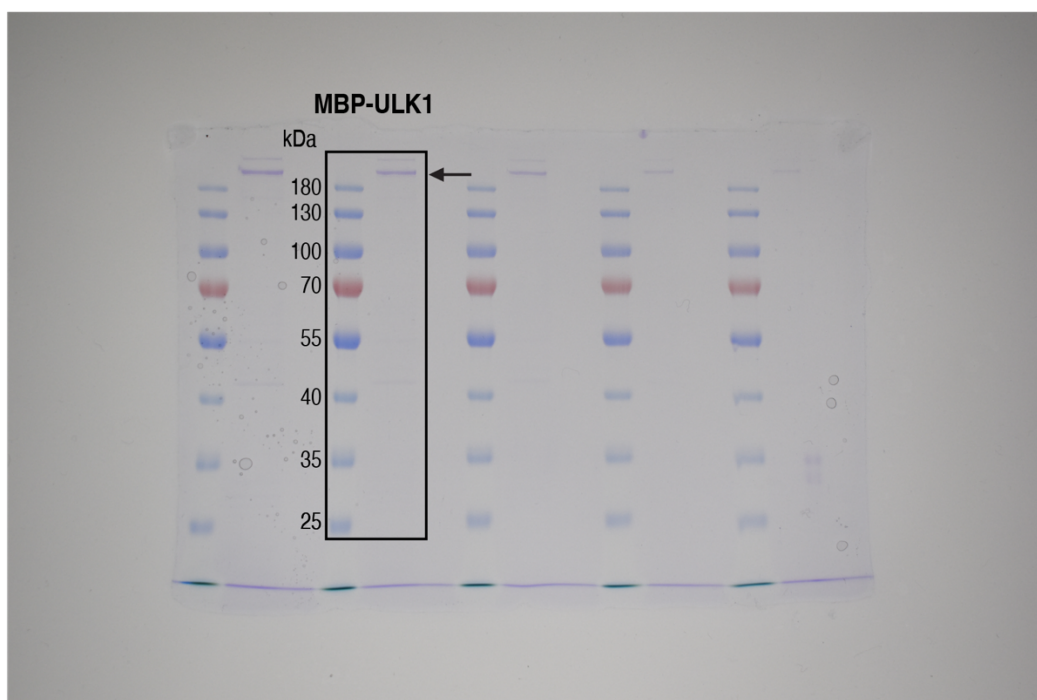

Figure S2D

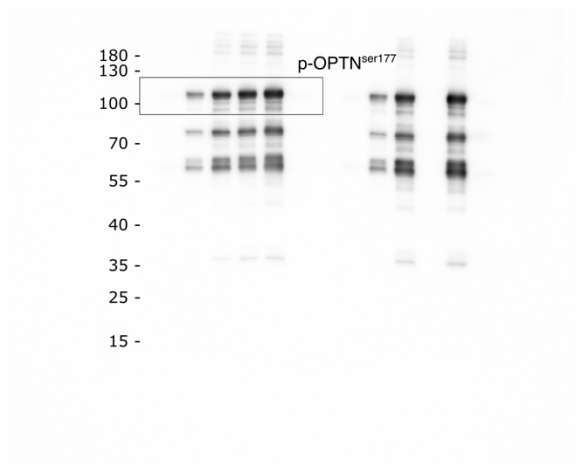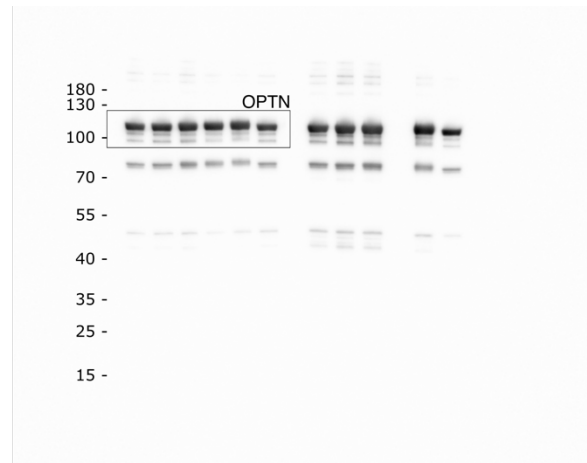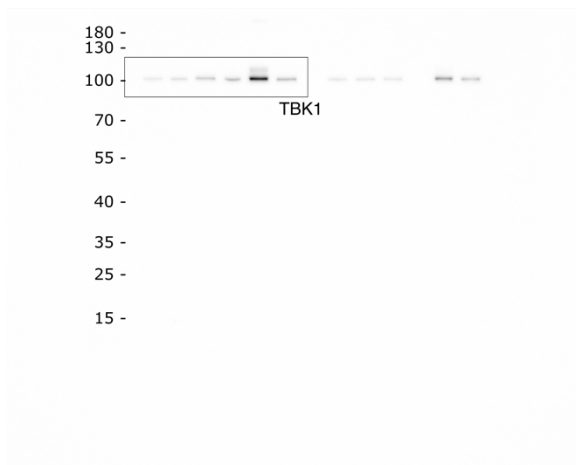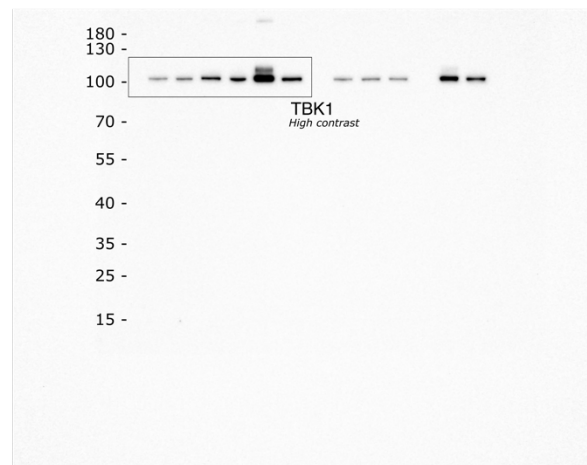

Figure S2E

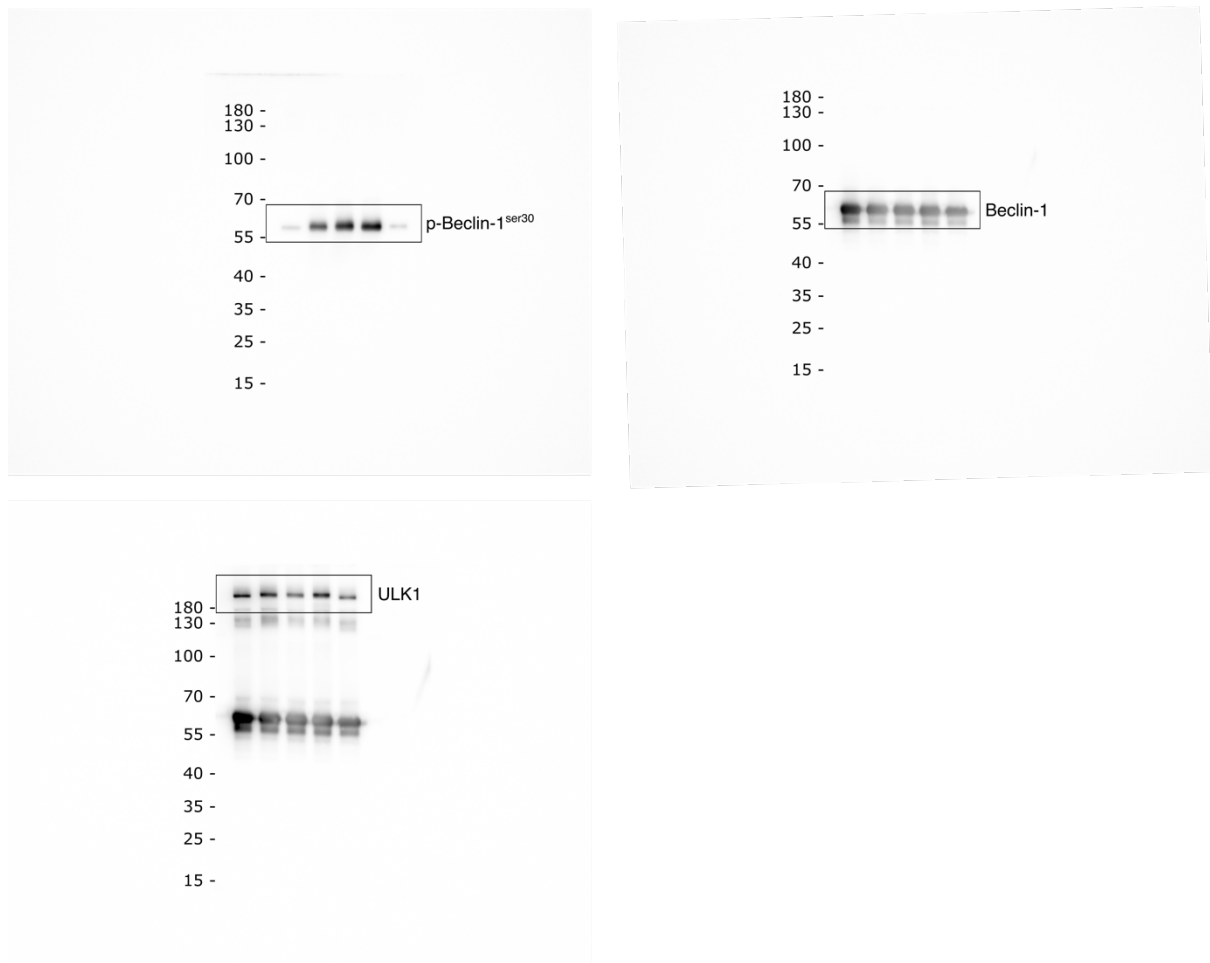

Figure S2F

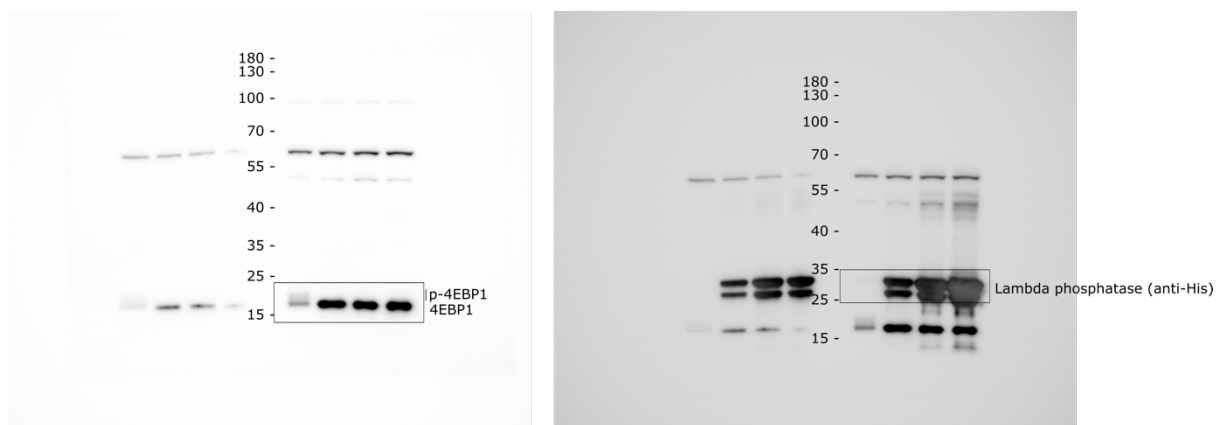

Supplement: Supplementary file 19 — Unprocessed gels and western blots [file 41556_2025_1712_MOESM19_ESM.pdf]

Figure S3B

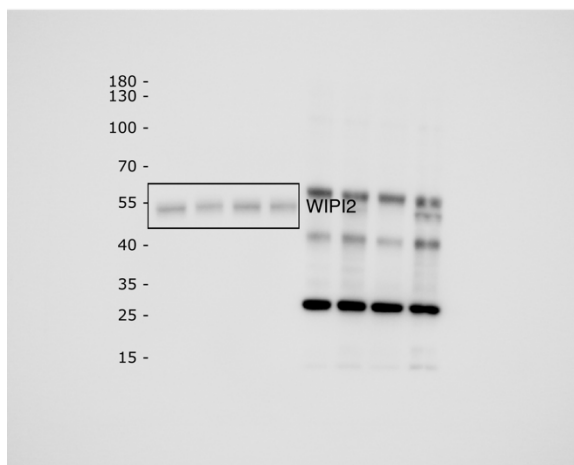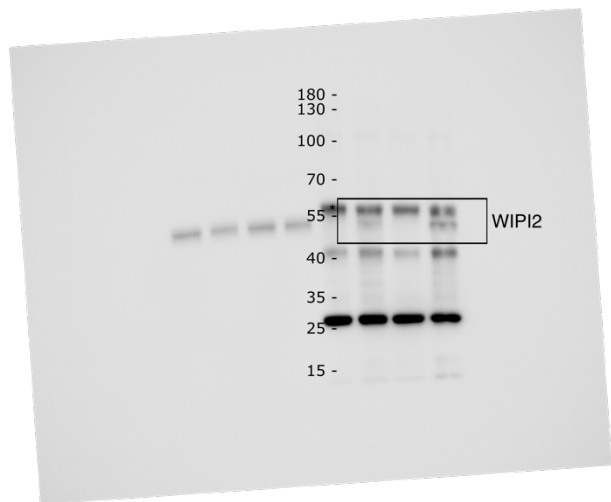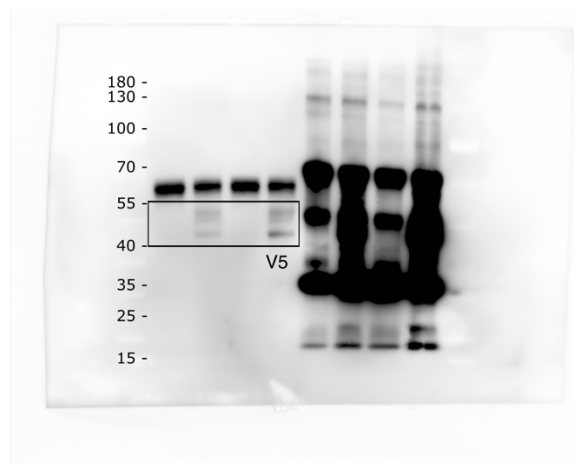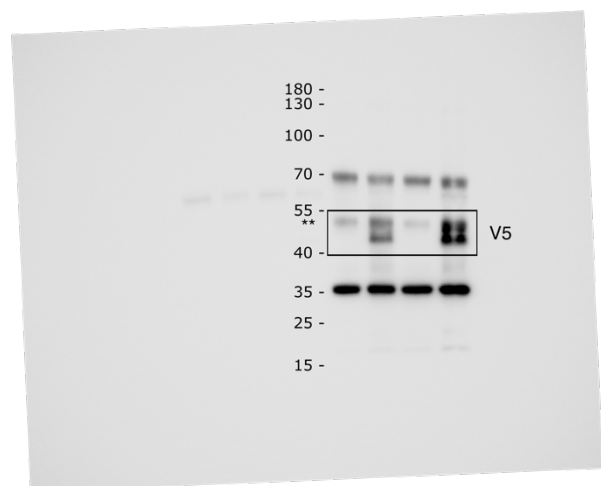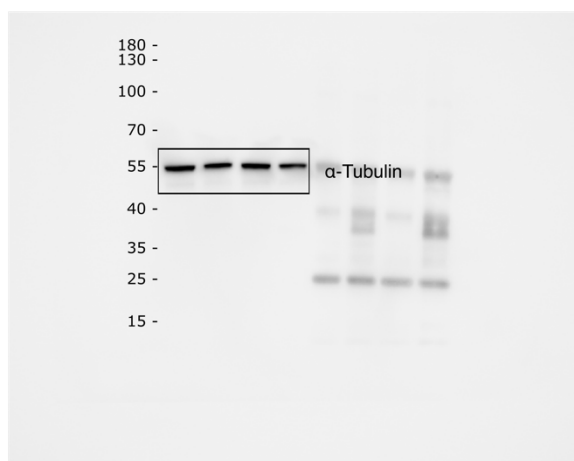

Supplement: Supplementary file 20 — Unprocessed western blots [file 41556_2025_1712_MOESM20_ESM.pdf]

Figure S5A

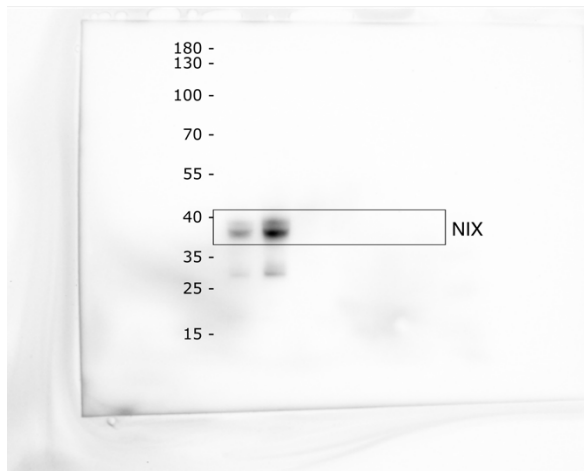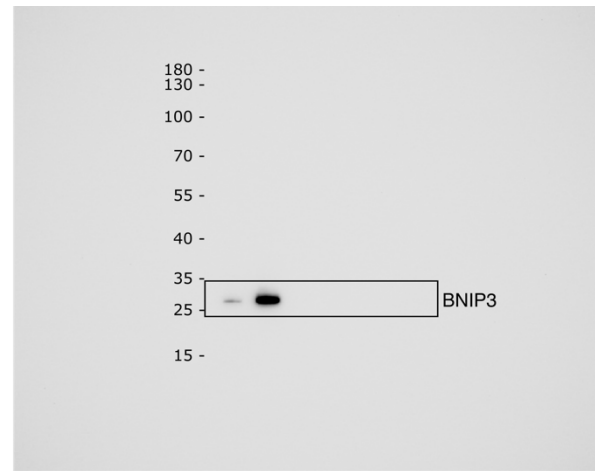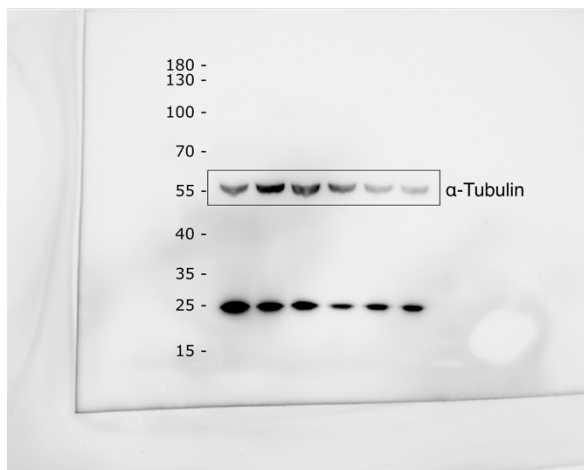

Figure S5C

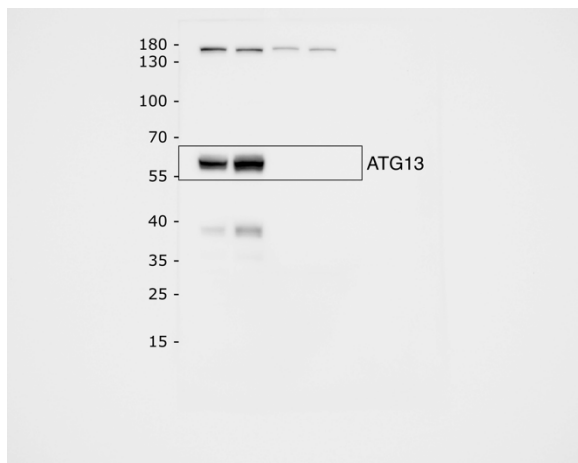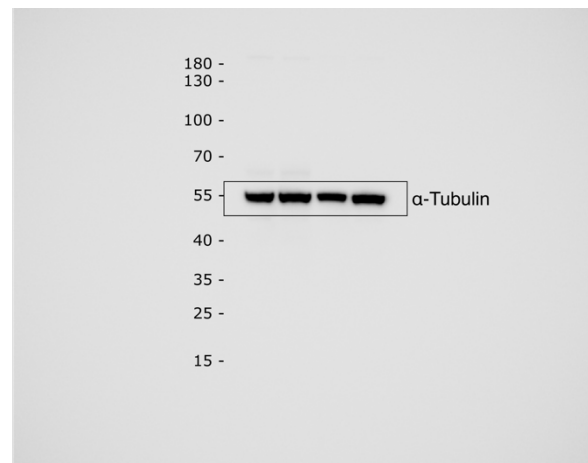

Figure S5D

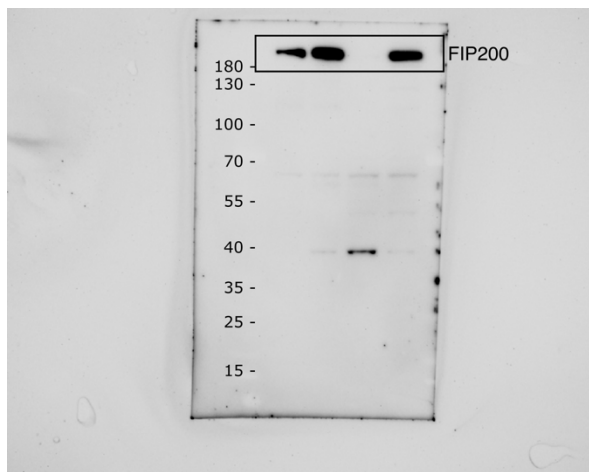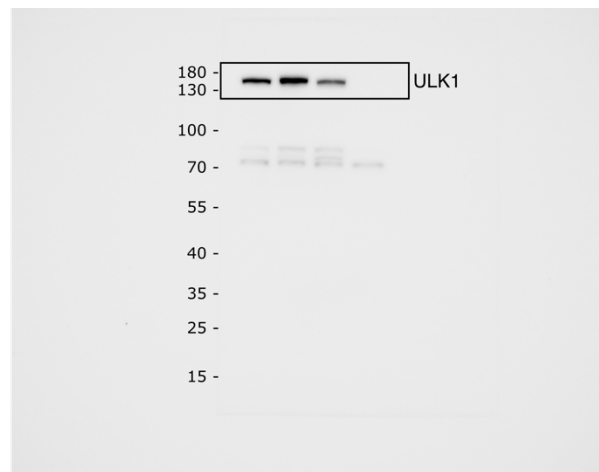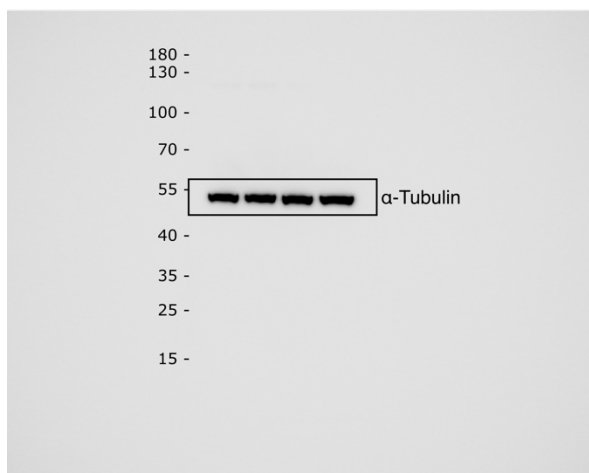

Supplement: Supplementary file 23 — Unprocessed western blots [file 41556_2025_1712_MOESM23_ESM.pdf]

Figure S8C

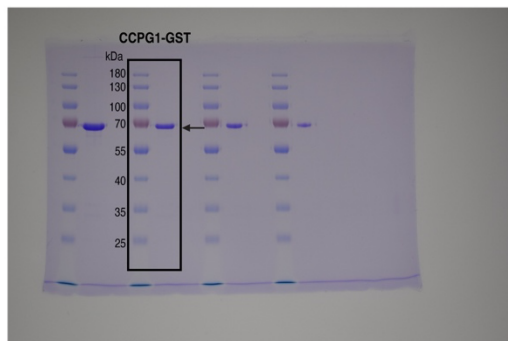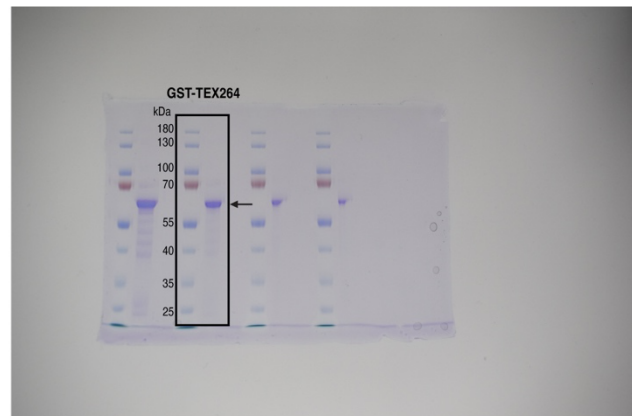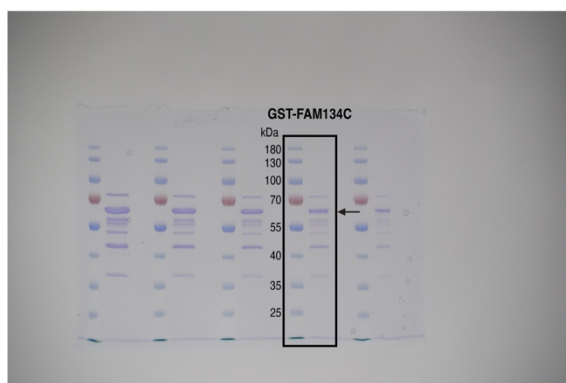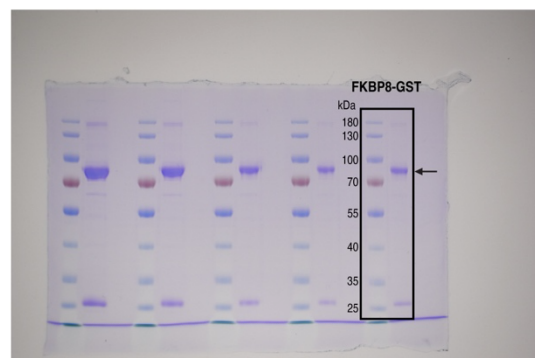

Supplement: Supplementary file 24 — Unprocessed gels [file 41556_2025_1712_MOESM24_ESM.pdf]
